# Diffuse large B-cell lymphoma presenting as a scalp tumor with skull destruction and neurological symptoms: a case report

**DOI:** 10.3389/fimmu.2026.1857412

**Published:** 2026-05-29

**Authors:** Ayaka Yasuda, Natsuko Saito-Sasaki, Hirofumi Kawamoto, Yu Sawada

**Affiliations:** Department of Dermatology, University of Occupational and Environmental Health, Kitakyushu, Japan

**Keywords:** bone, case report, diffuse large B - cell lymphoma, RANKL, scalp

## Abstract

Diffuse large B-cell lymphoma (DLBCL) frequently involves extranodal sites; however, extensive skull destruction and suspected central nervous system (CNS) involvement associated with a scalp lesion are extremely rare. A 70-year-old man presented with a rapidly enlarging scalp tumor. Histopathological examination revealed diffuse proliferation of large atypical B lymphocytes consistent with a non-GCB phenotype of DLBCL. Imaging studies demonstrated a large scalp mass with marked osteolytic skull destruction and extension into the superior sagittal sinus with leptomeningeal enhancement. Systemic evaluation revealed multi-organ involvement, including bone marrow infiltration, leading to a diagnosis of stage IV DLBCL with secondary cutaneous involvement. Immunohistochemical analysis demonstrated expression of receptor activator of nuclear factor-κB ligand (RANKL) in tumor cells. The patient was treated with Pola-R-CHP followed by CNS-directed therapy, including high-dose methotrexate and intrathecal chemotherapy. The tumor showed marked regression, accompanied by improvement of neurological symptoms. This case highlights a rare presentation of DLBCL with skull destruction and suspected CNS involvement. Tumor-associated RANKL expression may be associated with the osteolytic features observed in this case. Early biopsy and systemic evaluation of cutaneous lesions are essential, as they may reflect aggressive systemic disease.

## Introduction

Diffuse large B-cell lymphoma (DLBCL) is the most common subtype of non-Hodgkin lymphoma and frequently involves extranodal sites (1). Cutaneous manifestations of DLBCL may represent either primary cutaneous lymphoma or secondary involvement from systemic disease, and distinguishing between these entities is clinically important (2, 3). However, extensive skull destruction and suspected central nervous system (CNS) involvement presenting with a scalp lesion are extremely rare.

Here, we report a rare case of DLBCL presenting as a scalp tumor with marked osteolytic skull destruction and CNS involvement. Notably, immunohistochemical analysis revealed expression of receptor activator of nuclear factor-κB ligand (RANKL) in tumor cells, suggesting a possible association with tumor-associated bone destruction.

## Case presentation

**Figure 1:**
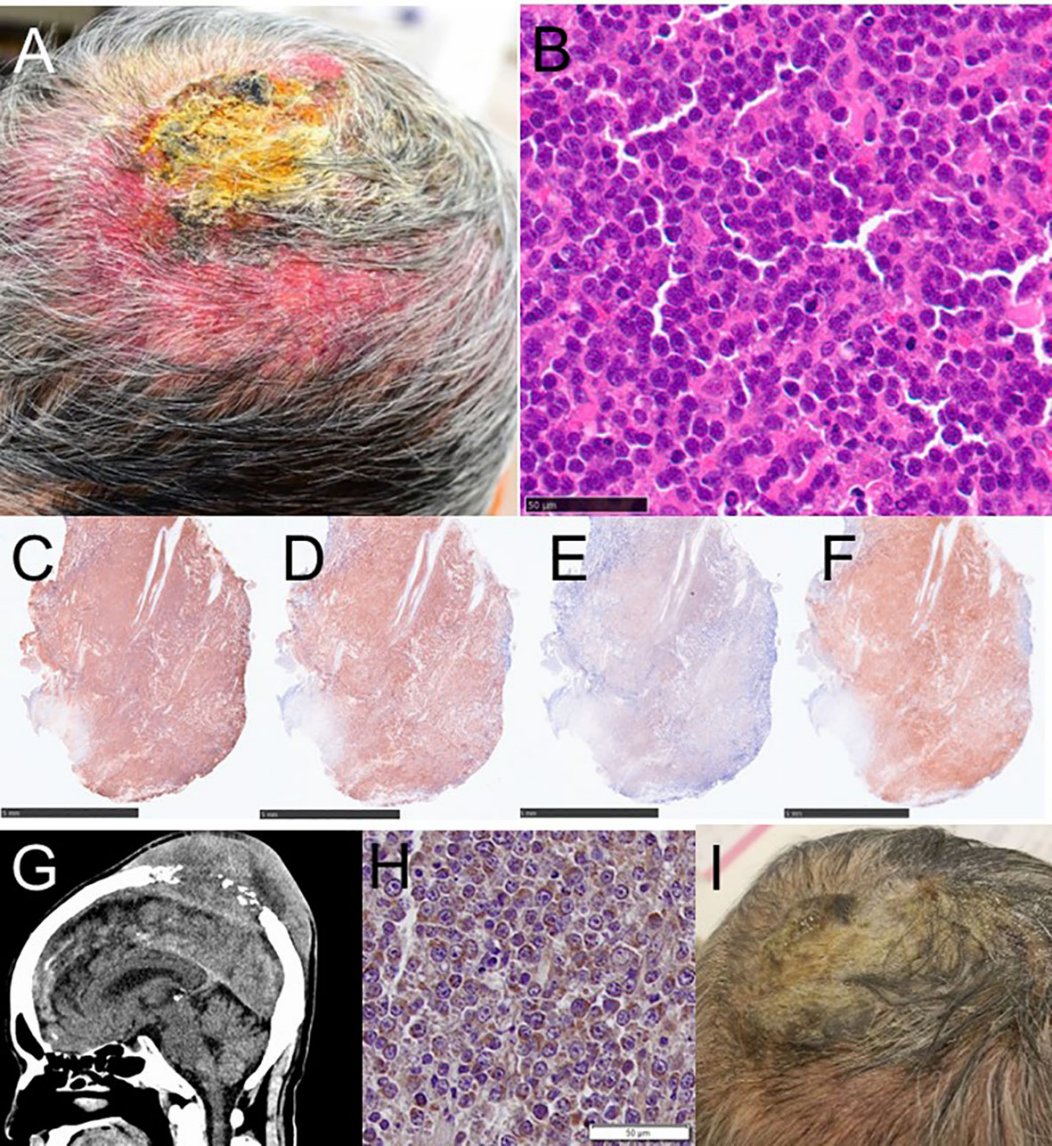
Clinical, histopathological, immunohistochemical, and radiological findings. **(A)** Clinical presentation showing an erythematous scalp lesion with yellow crust and erosion. **(B)** Hematoxylin and eosin staining showing diffuse proliferation of medium-to-large atypical lymphocytes extending from the dermis to the subcutaneous tissue (scale bar = 50 μm). **(C–F)** Immunohistochemical staining demonstrating positivity for CD20 **(C)**, BCL2 **(D)**, BCL6 **(E)**, and MUM1 **(F)** in tumor cells (scale bars = 5 μm). **(G)** Computed tomography showing a large soft tissue mass in the posterior scalp with osteolytic skull destruction (arrows). **(H)** Immunohistochemical staining for RANKL demonstrating positive expression in lymphoma cells (scale bars = 50 μm). **(I)** Clinical image after treatment showing marked regression of the scalp tumor.

A 70-year-old man presented with a progressively enlarging scalp skin tumor over six months. Physical examinations showed erythematous skin lesions with yellow crust and erosion on the scalp (**Figure 1A**). At initial presentation, differential diagnoses included cutaneous squamous cell carcinoma, metastatic tumor, angiosarcoma, and other lymphoid malignancies because of the rapidly enlarging scalp tumor with skull destruction. A skin biopsy taken from the erythematous lesion demonstrated proliferation of medium-to-large atypical lymphocytes extending from the dermis to the subcutaneous tissue (**Figure 1B**). Immunohistochemical analysis showed positivity for CD20, BCL2, BCL6, and MUM1 (**Figures 1C–F**), and negativity for CD3, CD5, CD10, and cyclin D1. MYC, CD30, and EBER staining, as well as FISH analyses for MYC/BCL2/BCL6 rearrangements, were not performed. The Ki-67 labeling index was approximately 80%. According to the Hans algorithm, these immunohistochemical findings were consistent with a non-GCB phenotype (ABC-like subtype) of DLBCL.

Biochemical examination revealed elevated lactate dehydrogenase (260 U/L), markedly increased soluble interleukin-2 receptor levels (5237 U/mL), and hypercalcemia (11.7 mg/dL). Computed tomography revealed a large soft tissue mass (56 × 133 × 124 mm) in the posterior scalp with osteolytic skull destruction (**Figure 1G**). Magnetic resonance imaging showed tumor extension into the superior sagittal sinus and contrast enhancement in the leptomeninges, suggesting CNS involvement. Cerebrospinal fluid examination did not reveal atypical lymphoid cells. However, MRI findings and recurrent episodes of right lower limb weakness raised suspicion of CNS involvement. Positron emission tomography-computed tomography demonstrated increased uptake in the scalp skin tumor, left palatine tonsil, right seminal vesicle, and ascending colon. Bone marrow examination revealed infiltration of atypical lymphocytes. Based on these findings, the patient was diagnosed with stage IV DLBCL involving the scalp, CNS, seminal vesicle, and bone marrow. Interestingly, immunohistochemical analysis additionally demonstrated positive expression of receptor activator of nuclear factor-κB ligand (RANKL) in the tumor cells (**Figure 1H**, **Supplementary Figure 1**).

Pola-R-CHP was selected because the patient had high-risk DLBCL with an IPI score of 4. This decision was supported by the POLARIX trial (4), which demonstrated improved progression-free survival with Pola-R-CHP compared with R-CHOP in previously untreated DLBCL patients with IPI ≥2. Given the advanced stage and clinically suspected CNS involvement, systemic chemotherapy with Pola-R-CHP was initiated every 21 days for six cycles. In addition, high-dose methotrexate (1.6 g/m², 2400 mg) was administered for six cycles, together with three courses of intrathecal chemotherapy consisting of methotrexate (10 mg), prednisolone (20 mg), and cytarabine (20 mg). Mild nausea and loss of appetite were observed during treatment. Soluble IL-2 receptor levels decreased significantly to 442 U/mL at 21 days after treatment initiation, and neurological symptoms, including lower limb weakness and numbness, gradually improved. A follow-up head MRI performed three months after the initiation of Pola-R-CHP demonstrated marked regression of the scalp mass, although mild dural thickening and residual abnormal bone signal enhancement persisted. Based on these clinical and radiological findings, the treatment response was assessed as partial response. The patient has remained clinically stable without evidence of disease progression during the currently limited 6-month follow-up period.

## Discussion

The present case is characterized by highly invasive local features at presentation, including extensive skull destruction and suspected CNS involvement. Such a presentation is extremely rare in DLBCL, particularly when initially manifested as a cutaneous tumor.

At diagnosis, multiple systemic lesions were identified, making it difficult to determine the primary site. The presence of widespread systemic involvement, including bone marrow and visceral organs, supported the diagnosis of systemic DLBCL with secondary cutaneous involvement rather than primary cutaneous lymphoma. Although the primary site could not be definitively determined, the scalp lesion was clinically dominant and was responsible for the most prominent local tissue destruction and neurological manifestations.

To our knowledge, RANKL expression has not been described in previously reported comparable cases. Immunohistochemical analysis demonstrated positive expression of RANKL in the lymphoma cells in our case. RANKL is a key regulator of osteoclast differentiation and activation and has been implicated in bone destruction in various malignancies (5, 6). In the present case, RANKL expression in tumor cells may suggest a possible association with the osteolytic features observed in this case; however, a causal relationship cannot be established from the present findings alone (7, 8). Although the biological significance of RANKL expression remains uncertain, these findings may provide insight into mechanisms associated with bone-destructive features in DLBCL.

We reviewed previously reported cases of DLBCL involving the cranial vault and scalp, focusing on tumor site, skull destruction, dural or leptomeningeal involvement, treatment, and clinical outcome (**Table 1**) (9–22).

**Table 1:** Reported cases of diffuse large B-cell lymphoma involving the cranial vault and scalp.

Author	Sex/Age	Tumor site	Skull destruction	Dural or leptomeningeal involvement	Treatment	Outcome
Our case	M/70	Posterior neck	Yes	Leptomeningeal involvement	Pola-R-CHP 6 courses plus HD-MTX	Alive
Kumarasamy S, et al. (9)	F/25	Right frontal scalp	Yes	Leptomeningeal involvement	Right frontal craniotomy	Died
Matejka M, et al. (10)	F/67	Left parietal scalp	No	Leptomeningeal involvement	Tumor resection, MATRix-protocol chemotherapy	Alive
Nishimoto T, et al. (11)	M/63	Left parietal region	No	No	CHOP chemotherapy	Alive
Ochiai H, et al. (12)	M/72	Left temporoparietal region	No	No	Whole-brain radiation therapy, CHOP chemotherapy	Alive
Davis J, et al. (13)	M/69	Left periauricular and temporal areas	Yes	Dural involvement	R-CHOP chemotherapy	Alive
Jha VC, et al. (14)	M/53	Posterior cranial vault	Yes	Dural involvement	Gross total excision, CHOP chemotherapy, Local adjuvant external beam radiotherapy	Alive
Umemura T, et al. (15)	F/72	Left parietal region	Yes	Dural involvement	Tumor resection, Whole-brain radiation therapy	Alive
Umemura T, et al. (15)	M/63	Occipital region	Yes	Dural involvement	Partial tumor resection, CHASER chemotherapy	Alive
King ML et al. (16)	M/40	Left frontoparietal region	No	No	R-EPOCH, Radiation therapy	Alive
Jaiswal M et al. (17)	F/40	Bilateral frontoparietal region	Yes	Dural involvement	Wide excision	N.D
Lv ZW et al. (18)	M/56	Parietal-occipital area	Yes	Leptomeningeal involvement	Tumor resection, CHOP chemotherapy	Alive
De Unamuno Bustos B, et al. (19)	M/85	Diffuse infiltration on the scalp	No	No	CHOP chemotherapy	Died
Kosugi S, et al. (20)	F/82	Right parietal area	Yes	Dural involvement	Radiotherapy plus R-CHOP	Alive
Rezaei-Kalantari K, et al. (21)	M/42	Left frontal head	Yes	Dural involvement	CHOP chemotherapy	Alive
Mundi JP, et al. (22)	M/56	Forehead and frontal aspect of the scalp, Left neck	N.D	N.D	N.D	N.D
Ochiai H, et al. (12)	M/72	Left temporoparietal scalp	No	Dural involvement	CHOP chemotherapy	Alive

MATRix-protocol, rituximab/HD-methotrexate/thiotepa/cytarabine; N.D, Not determined.

Several cases showed skull destruction and dural involvement, whereas leptomeningeal involvement was less frequently reported. Compared with these cases, the present case was notable for a clinically dominant scalp tumor with marked skull destruction, suspected leptomeningeal involvement, and extensive systemic disease including bone marrow and visceral organ involvement.

A limitation of this case report is the relatively short follow-up period of 6 months, which precludes a definitive assessment of the long-term disease course and treatment response. Further clinical follow-up is required to evaluate the durability of the therapeutic response.

Clinically, this case highlights the importance of early biopsy and prompt systemic evaluation, as cutaneous lesions in DLBCL may occasionally indicate deeply invasive or advanced systemic disease.

## Conclusion

We report a rare case of DLBCL presenting as a scalp tumor with skull destruction and neurological manifestations. Early biopsy of suspicious skin lesions is crucial for prompt diagnosis and treatment initiation, particularly in aggressive cases with systemic involvement. Although the biological significance of RANKL expression remains uncertain, the present case may provide insight into mechanisms associated with bone-destructive features in DLBCL.

## Data Availability

The original contributions presented in the study are included in the article/**Supplementary Material**. Further inquiries can be directed to the corresponding author.

## Supplementary material

Supplementary Figure 1 Negative control immunohistochemical staining for RANKL. Negative control staining was performed using the same RANKL staining protocol without the primary antibody.
