# Supplementary figures and images for: Diffuse large B-cell lymphoma presenting as a scalp tumor with skull destruction and neurological symptoms: a case report
Source: Front Immunol. 2026 May 29;17:1857412. doi: 10.3389/fimmu.2026.1857412 (PMC13257942; doi:10.3389/fimmu.2026.1857412)

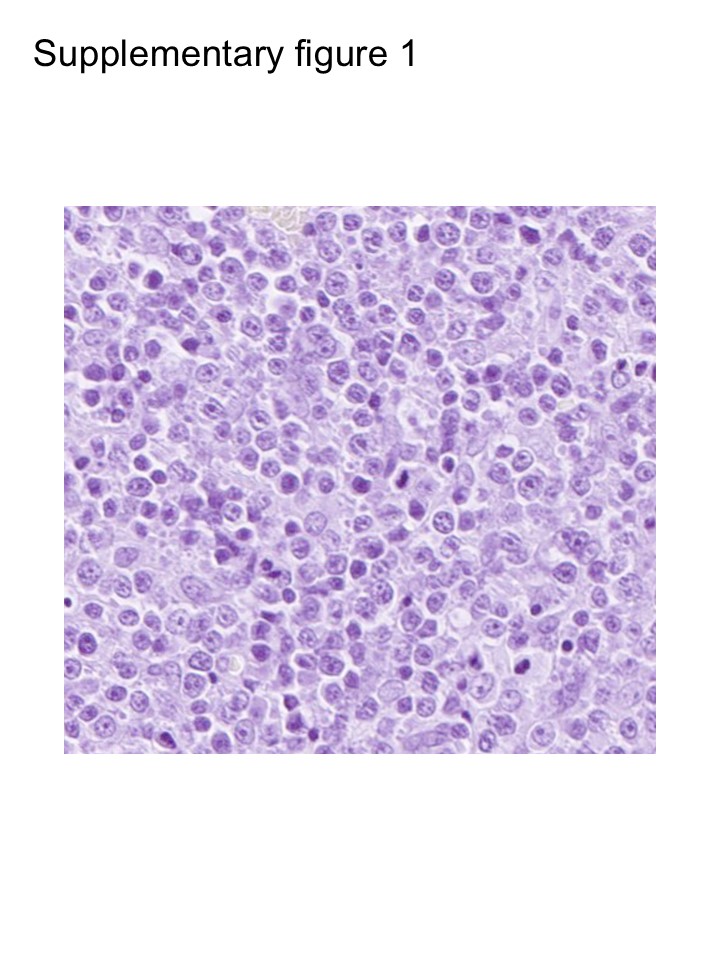

Supplement: Supplementary Figure 1 — Negative control immunohistochemical staining for RANKL. Negative control staining was performed using the same RANKL staining protocol without the primary antibody. [file Image1.jpeg]
